# Investigating the Effects of Dietary Bile Acids on Production Performance and Lipid Metabolism in Late-Phase Laying Hens

**DOI:** 10.3390/ani14243554

**Published:** 2024-12-10

**Authors:** Longfei Wang, Kefeng Fan, Ronghui Xing, Jixue Yin, Xuemeng Si, Huaiyong Zhang, Yanqun Huang, Wen Chen

**Affiliations:** 1 Institute of Animal Science and Technology, Henan Agricultural University, Zhengzhou 450046, China; wlongfei2024@outlook.com (L.W.); x2044280462@outlook.com (R.X.); jiixue@163.com (J.Y.); sxmswun@126.com (X.S.); huaiyong.zhang@ugent.be (H.Z.); hyanqun@aliyun.com (Y.H.); 2 College of Animal Science and Technology, Henan University of Animal Husbandry and Economy, Zhengzhou 450046, China; fanke333@aliyun.com; 3 Laboratory for Animal Nutrition and Animal Product Quality, Department of Animal Sciences and Aquatic Ecology, Ghent University, 9000 Ghent, Belgium

**Keywords:** bile acids, late-phase laying hens, lipid metabolism, laying performance

## Abstract

Multiply adverse effects including declines in production performance and excessive fat deposition were noticed with the extension of the laying cycle in hens, which are pertinent to animal welfare and human food safety. This study aimed to investigate the effect of dietary supplementation of bile acids (BAs) on production performance and lipid metabolism in late-phase laying hens. A total of 144 70-week-old hens were distributed into three treatments with eight replicates per treatment, including the basal diet with 0 (Ctrl), 95.01 (Low-BA), and 189.99 mg/kg (High-BA) of porcine BAs, respectively. The test period was from 70 to 75 weeks. The supplementation of BAs did not significantly alter laying performance during the trial, whereas it increased (*p* < 0.05) the total follicles compared to the Ctrl diet. The eggs from the hens fed the BA diet exhibited increased (*p* > 0.05) relative weight of eggshell and yolk color than those that consumed the Ctrl diet. There were no significant changes following BA treatment regarding the serum lipid profile. Dietary BA treatment reduced the total triglyceride in livers to different extents, resulting in the decreased diameter and area of vacuoles in liver tissues. The low-dose BA treatment decreased the mRNA levels of fatty acid synthase (*FASN*) and stearoyl-CoA desaturase (*SCD*), while promoting the expression of lipoprotein lipase (*LPL*) compared to the Ctrl group (both *p* < 0.05). Of note, the expressions of farnesoid X receptor (*FXR*), apical sodium-dependent bile acid transporter (*ASBT*), and ileum bile acid-binding protein (*IBABP*) were notably downregulated (*p* < 0.05) by the low-dose BA treatment. Dietary BA treatment had no apparent effects on laying performance, whereas it increased the follicle frequency, eggshell weight, and yolk color. Moreover, a diet containing 95.01 mg/kg of BAs depressed ileal BA resorption and hepatic fatty deposition by reducing lipogenesis and promoting lipolysis, which may have a beneficial effect on the liver in late-phase layers.

## Simple Summary

In the current study, the effect of dietary bile acids on production performance and lipid metabolism in late-laying hens was evaluated. The outcome showed that dietary supplemented with porcine-derived bile acids had no significant effect on production performance but increased total follicle number, relative eggshell weight, and yolk color. Of note, 95.01 mg/kg porcine-derived bile acids inhibited ileal bile acid reabsorption and hepatic lipid deposition by inhibiting fat synthesis and promoting lipolysis. These data suggest that bile acids improve egg quality and play a key role in lipid metabolism in laying hens.

## 1. Introduction

During the extension of the laying cycle in hens, adverse effects have been observed, including declines in egg production, deterioration in egg quality, and the incidence of fatty liver hemorrhagic syndrome (FLHS) [1], which are pertinent to animal welfare and human food safety. Of note, the prevalence rate of FLHS is as high as 16%, which is induced by the excessive accumulation of liver and abdominal fat and causes severe hepatic steatosis and liver bleeding [2,3]. It is reported that FLHS leads to significant reductions in egg production and is responsible for more than 74% of non-infectious deaths in caged laying hens [4]. One of the pathogenic mechanisms is the excess deposition of fatty acids, resulting in excessive lipogenesis, increased fat transfer, and reduced lipolysis. This would lead to lipid droplets in the hepatic cytoplasm pushing the nucleus of hepatocyte against the cell membrane, indicating fatty degeneration of the organ [5]. Increasing evidence has shown the possibility that hepatic lipid accumulation may evoke oxidative stress and chronic inflammation, impairing hepatocellular function and triggering apoptosis [6]. Therefore, it is possible that reducing hepatic lipid deposition might improve hepatic health status in post-peak laying hens [7].

Bile acids (BAs), a group of hydroxylated steroids synthesized from cholesterol in the liver, have been widely used as fat emulsifiers in animal feed and play an important role in lipid metabolism [8]. The conversion of cholesterol to BAs for bile secretion is the major pathway for eliminating excess cholesterol [9]. Upon food ingestion, the gallbladder releases BAs into the duodenum to aid in the digestion of dietary lipids, which ultimately pass through the small intestine and colon [10]. Approximately 95% of BAs are reabsorbed in the terminal ileum and transported back to the liver through the portal vein [11]. Another 5% of BAs are excreted in feces. BAs are also important signaling molecules that are involved in glucose metabolism, lipid metabolism, and energy consumption [12]. BAs were discovered to be endogenous ligands of farnesoid X receptor (*FXR*) [13], which is widely expressed in the intestine, liver, and kidney. As a bridge between the liver and small intestine, *FXR* is highly expressed in both organs and helps maintain the homeostasis of cholesterol/bile acids by regulating various metabolic enzymes and transport proteins [14]. In addition, intestinal FXR regulates hepatic cholesterol 7-alpha-monooxygenase or cytochrome P450 7A1 (*CYP7A1*) through a mechanism dependent on fibroblast growth factor 15 (*FGF15*) [15]. Polin et al. found that the addition of 0.04% BAs in the diet improved the absorption of fat in chicks and the digestibility and efficiency of dry matter [16]. Dietary supplementation with 0.08 g/kg BAs reduced serum triglyceride (TG) levels and abdominal fat deposition by upregulating the expressions of lipolytic gene and reducing lipogenic gene expression [17]. In laying hens, studies have shown that supplementation of 60 mg/kg porcine bile acids increased the laying performance of late-phase hens, and a diet containing 60 and 90 mg/kg of bile acids decreased serum TG, total cholesterol (TC), and low-density lipoprotein cholesterol (LDL-c), leading to reduced abdominal adipose tissue [18]. However, Sun et al.’s research results showed that 100 mg/kg and 200 mg/kg BAs had no significant effect on the production performance and serum lipid metabolism of laying hens but reduced liver lipid deposition and mortality [19]. Moreover, the protective roles of BAs were also noticed in laying hens that received the high-fat diet (HFD), evidenced by improving laying performance and egg quality and reversing liver disease [20]. These findings suggest that dietary BA supplementation might improve the production performance and lipid metabolism of late-phase laying hens.

In practice, the laying cycle in commercial laying hens lasts from 72 weeks up to 80 weeks [21], which might aggravate the decrease in production performance and egg quality, dyslipidemia, and hepatic impairment in late-phase laying hens. Accordingly, the purpose of this study is to investigate the effect of dietary porcine BA supplementation on production performance and lipid metabolism in laying hens from 70 to 75 weeks. Overall, the innovation in this study is primarily related to the use of BAs as a dietary supplement to improve lipid metabolism and specific egg quality factors in late-phase laying hens without negatively affecting their production performance, which provides a theoretical basis and data support for prolonging the producing period and improves the liver health of layers.

## 2. Materials and Methods

### 2.1. Birds, Diet, and Management

After 2 weeks of feeding for gradual acclimation, a total of 144 healthy 70-week-old Hy-line Brown layers with a similar laying rate were selected for this study in Feng yuan Poultry Co., Ltd. (Nanyang, China) and allocated into 3 treatments with 8 replicates per treatment of 48 hens, including the control group (Ctrl, feeding the basal diet), low dose of BAs group (Low-BA), and high dose of BAs group (High-BA). The BAs used in this study were obtained by Jiuyi Chinese Medicine Research Institute (Zhengzhou, China) and derived from the gallbladders of pigs. The levels of BAs in Low-BA and High-BA groups were analyzed by high-performance liquid chromatography and included 95.01 and 189.99 mg/kg, respectively. The basal corn/soybean diet was formulated according to the China Agricultural Industry Standards (2004) [22], as shown in Table 1. The diet was provided in powder form, and all birds could freely obtain water and food during the experiment. The light schedule was 16 h and dark for 8 h per day (16L:8D), according to normal management practices. The temperature and relative humidity in the house were kept at around 25 °C and 65%, respectively. Artificial insemination was performed at five-day intervals. Disinfection and epidemic prevention measures are all carried out following the routine management plan of the poultry farm.

### 2.2. Laying Performance

During the experiment, eggs were collected daily from each replicate, and the number and weight of eggs were recorded. The daily egg production rate (%) was calculated by dividing the number of eggs per day per replicate by the number of birds. Average egg weight was calculated by dividing the total daily egg weight per replicate by the number of eggs. Egg mass was derived by multiplying the egg production rate (%) by the average egg weight (g). Feed intake was recorded weekly and daily feed intake (ADFI) was calculated for each replicate. The feed conversion ratio (FCR) was determined by dividing the feed consumption by the total egg weight per replicate.

### 2.3. Egg Quality

At 75 weeks of age, 6 fresh eggs were collected for egg quality determination in each replicate. After weighing, the length and width of the egg were measured using a vernier caliper, and the egg shape index expressed as the ratio of egg length to egg width was calculated. Eggshell strength was determined with an eggshell strength meter (EFG-0503, Robotmation Co., Ltd., Tokyo, Japan). Subsequently, the eggshell was separated, and its relative weight was obtained from the weight of the eggshell and the whole egg. The thickness of the eggshell is determined by an eggshell thickness tester (ETG-1061, Robotmation Co., Ltd., Tokyo, Japan) based on the average thickness of the eggshell tip, middle part, and blunt end. Haugh unit (HU), yolk color, and protein height were measured using an Automatic egg quality tester (EMT-5200, NABEL Co., Ltd., Kyoto, Japan). Finally, the weight, length, and width of egg yolk were registered to calculate the relative weight and yolk index according to our previous method.

### 2.4. Sample Collection and Organ Indexes

At 75 weeks of age, one hen was selected from each pen for weighting and sampling after a 12 h fast. Blood was collected from a sub-wing vein and centrifuged in a centrifuge at 3000 rpm at 4 °C for 10 min to obtain serum. The heart, liver, spleen, intestine, pancreas, and ovaries were dissected and weighed after euthanizing. The corresponding relative weight was calculated by dividing by the body weight. Moreover, the ratios of intestinal length to body weight defined as the relative length of the intestine were also obtained for duodenum, jejunum, and ileum. Of note, the follicles were separated from the ovaries and measured with a ruler; diameters of 3–5 mm and greater than 6 mm were classified as white follicles and yellow follicles, respectively. The left liver tissue samples were obtained and immediately snap-frozen in liquid nitrogen and stored at −80 °C for determination of hepatic lipid metabolism-related gene expression. A part of the right liver tissue was fixed with 4% formaldehyde for morphological analysis. Ileal mucosa was collected from the middle segment of the ileum and immediately stored at −80 °C for subsequent gene expression measurement.

### 2.5. Serum and Hepatic Lipid Accumulation

The levels of serum TG, TC, LDL-c, and high-density lipoprotein cholesterol (HDL-c) were determined by using reagent kits by the manufacturer’s protocols. The hepatic contents of TG, TC, HDL-c, and LDL-c were measured according to the instructions of the commercial assay kits. In detail, the liver was diluted by anhydrous ethanol as 1 g: 9 mL for grinding. The liver grinding fluid was centrifugated at 2500 rpm/10 min at 4 °C, and the supernatant was collected for determination of TG, TC, HDL-c, and LDL-c. All measurements of each variable were run in the same assay to avoid intra-assay variability. The results of the parameters were calculated as millimole per gram protein (mmol/g). All kits were obtained from Nanjing Jiancheng Bioengineering Institute (Nanjing, China). In addition, the liver samples fixed with 4% paraformaldehyde solution were dehydrated, embedded, cut into cross-sections, and stained with Oil Red O to observe liver lipid deposition. All liver cross-sections were quantified by FIJI Image J (National Institutes of Health, Bethesda, MD, USA).

### 2.6. Assessment of Liver Injury

Fixed liver samples were processed with paraffin, and then cut into 4 mm. Hematoxylin and eosin (H&E) staining was performed using standard procedures. The area and diameter of hepatic vacuoles were quantified by Image-Pro Plus (IPP 6.0, Cyber Medianetics). The distribution histogram of lipid droplet diameter was determined by Origin Pro (OriginLab Corporation, Massachusetts, USA). Moreover, the levels of serum aspartate aminotransferase (AST) and alanine transaminase (ALT) were qualified using an automatic biochemistry analyzer (Chemray 800, Biobase Co., Ltd., Jinan, China).

### 2.7. Gene Expression Assays

Total RNA was separated from liver and ileum mucosa samples with TRIZOL reagent (TransGen Biotech Co., Ltd., Beijing, China) based on the manufacturer’s instructions. The concentration (ng/µL) and purity (OD 260/280) of the extracted total RNA were determined by using a spectrophotometer (Nanodrop 2000; Thermo Fisher Scientific, Waltham, MA, USA). The quality of RNA was determined using agarose and acrylamide gel electrophoresis. After adjusting the concentration of total RNA to 1000 ng/µL, RNA was reversed into cDNA under the following conditions: 37 °C for 15 min, 85 °C for 5 s, and 4 °C for storage. The obtained cDNA was used subsequently to determine the expression of interest genes on the platform using ABI 7900 fluorescent quantitative polymerase chain reaction (Applied Biosystems, Warrington, UK) with SYBR qPCR Master Mix (Vazyme Biotech Co., Ltd., Nanjing, China). The reaction conditions were as follows: pre-denaturation 95 °C/30 s, 35 cycles (94 °C/5 s, 60 °C/34 s), and melting stage (95 °C/15 s, 60 °C/1 min, 95 °C/15 s). Primers were designed by NCBI (https://www.ncbi.nlm.nih.gov/ (accessed on 9 September 2024)) and are shown in Table 2. *β-actin* was used as the internal control to normalize the target gene transcript levels.

### 2.8. Statistical Analysis

The obtained data were analyzed by the Kolmogorov–Smirnov and Levene’s tests to assess normal distribution and homogeneity of variances, respectively, using SPSS 26 (SPSS Inc., Chicago, IL, USA). One-way analysis of variance (ANOVA) with Tukey’s test for normal distribution and Kruskal–Wallis test for non-normal distribution data were performed to evaluate the effects of BAs on the production performance, lipid metabolism, and liver health of late-phase laying hens, respectively. The statistical model was as follows:

*Y_j_* = *μ* + *D_j_* + *ε_j_*

Here, *Y_j_* is the average of treatment *j*, *µ* is the overall average, *D_j_* is the fixed effect of treatment *j*, and *ε_j_* is the error term. *p* < 0.05 and *p* < 0.1 were defined as statistically significant and tendency, respectively.

## 3. Results

### 3.1. Effects of BA on Laying Performance

**Figure 1:**
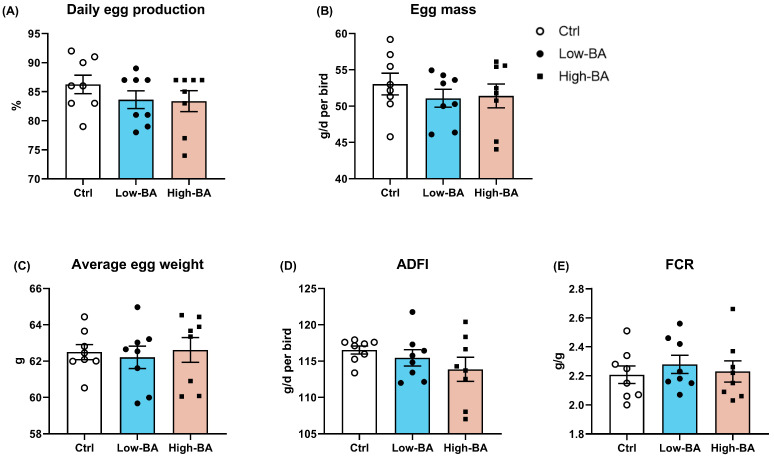
Effects of dietary bile acid (BA) supplementation on production performance in late-phase laying hens. (**A**) Daily egg production, (**B**) egg mass, (**C**) average egg weight, (**D**) average daily feed intake (ADFI), and (**E**) feed conversion ratio (FCR). Egg mass was derived by multiplying the egg production rate (%) by the average egg weight (g). FCR was determined by dividing the feed consumption by the total egg weight per replicate. *p* < 0.05 was set as statistically significant (*n* = 8).

**Figure 2:**
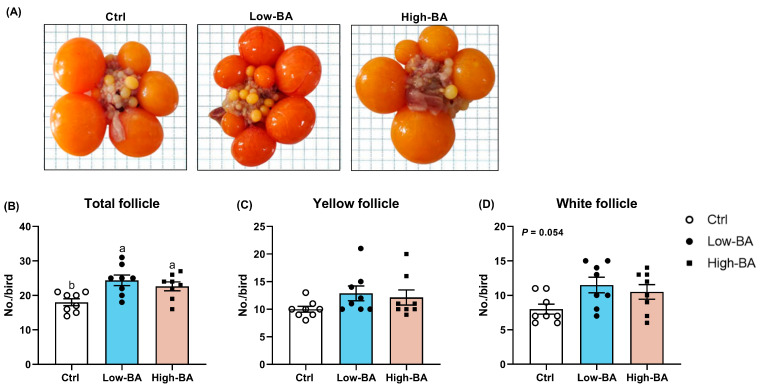
Effects of dietary bile acid (BA) treatments on number of follicles in late-phase laying hens (n = 10) (**A**) Representative image of ovarian follicles. The number of (**B**) total follicles including (**C**) pre-grade yellow follicles (diameter 6–12 mm) and (**D**) pre-grade white follicles (diameter 1–6 mm). ^a,b^ Different letters represent significant differences at *p* < 0.05 (*n* = 8).

The effect of BA supplementation on the laying performance is presented in Figure 1. There were no significant alterations (*p* > 0.05) regarding daily egg production, egg mass, average egg weight, ADFI, and FCR among Ctrl, Low-BA, and High-BA groups. Evaluating the number of follicles suggested that dietary BA treatment increased the total number of follicles compared to the Ctrl diet (*p* < 0.05), along with an increased number of yellow follicles (*p* > 0.05) and white follicles (*p* = 0.054) in the ovarian of late-phase laying hens (Figure 2).

### 3.2. Effects of BA on Egg Quality

As presented in Table 3, at 75 weeks of age, dietary BA supplementation increased the relative weight of eggshell and yolk color compared to the Ctrl group, especially the low dose of BA treatment in the diet of the layer. There were no apparent changes (*p* > 0.05) in terms of the egg-shaped index, eggshell strength, relative weight of yolk, and Haugh unit among the three groups (Table 3).

### 3.3. Effects of BA on Organ Indexes

The dietary BA treatment had no significant impact on the absolute and relative weight of the heart, liver, spleen, pancreas, and ovarian in the 75-week-old layer (Table 4). Although no significant difference in the absolute length of the small intestine was observed, the relative length of the jejunum in laying hens was increased (*p* = 0.070) with an increasing dosage of dietary BAs. Additionally, the relative length of the ileum in the Low-BA group was significantly lower compared to the High-BA birds, which was comparable to the Ctrl group (*p* < 0.05; Table 4).

### 3.4. Effects of BA on Liver Injury 

**Figure 3:**
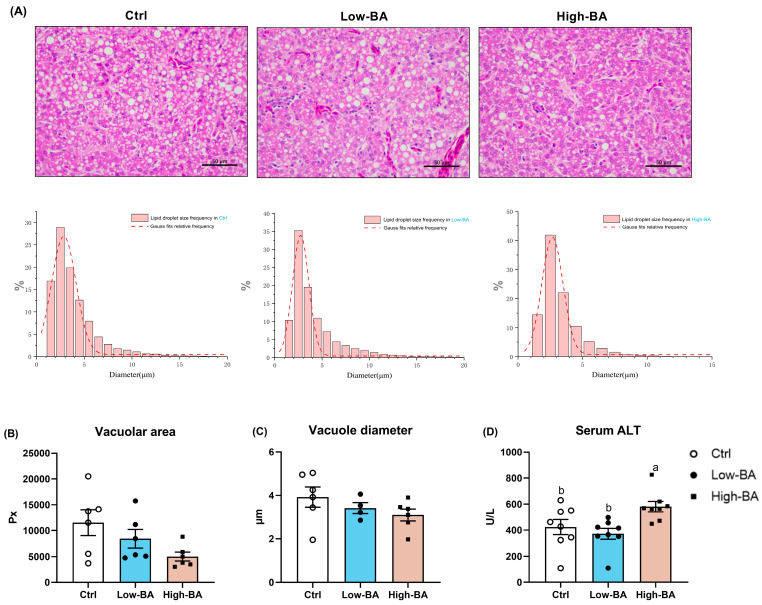
Effects of dietary bile acid (BA) supplementation on liver injury in late-phase laying hens. (**A**) Hematoxylin/eosin staining of the liver structure and the frequency distribution of diameter of white lipid droplet vacuoles, scale bar = 50 μm. (**B**,**C**) The area and diameter of white lipid droplet vacuoles (*n* = 6). (**D**) The content of serum alanine transaminase (ALT) in serum (*n* = 8). ^a,b^ Different letters represent significant differences at *p* < 0.05.

Figure 3A showed the presence of lipid droplet vacuoles in all treatment groups, accompanied by the compression of hepatocyte nuclei, according to H&E staining. The supplementation of BAs increased the particle size distribution from 2 to 4 μm in the Ctrl group to 3–5 μm in both the Low- and High-BA groups, respectively (Figure 3A). The analysis indicated that both the diameter and area of vacuoles tended to decrease as the BAs levels increased (Figure 3B,C). Moreover, a high dietary BA treatment notably elevated serum ALT levels compared to Ctrl and Low-BA groups (*p* > 0.05, Figure 3D).

### 3.5. Effects of BA Serum and Liver Lipid Levels

**Figure 4:**
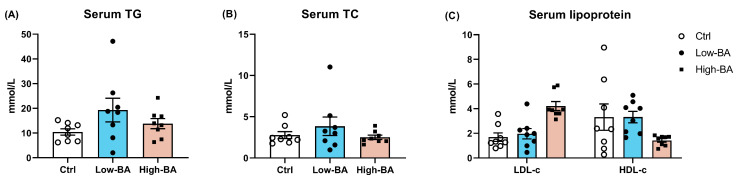
Effects of dietary bile acid (BA) treatments on serum lipid profile in late-phase laying hens. (**A**) The content of serum total triglycerides (TG), (**B**) total cholesterol (TC), and (**C**) low-density lipoprotein cholesterol (LDL-c) and high-density lipoprotein cholesterol (HDL-c). *p* < 0.05 was set as statistically significant (*n* = 8).

**Figure 5:**
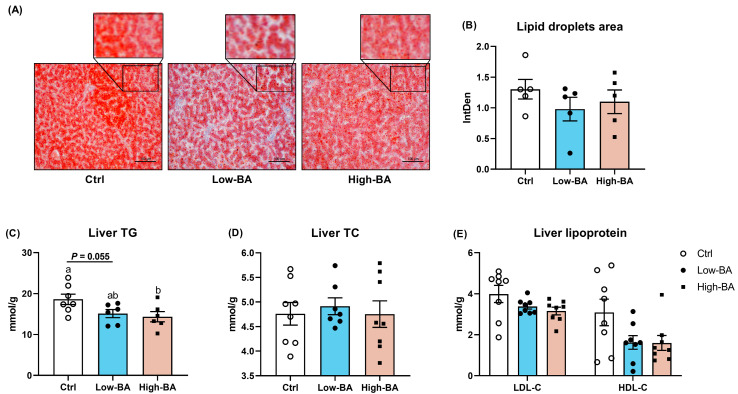
Effects of dietary bile acid (BA) supplementation on hepatic lipid deposition in late-phase laying hens. (**A**) Oil Red O staining of liver tissue, scale bar = 100 μm. (**B**) Lipid droplet area (*n* = 5). (**C**–**E**) The content of total triglycerides (TG), total cholesterol (TC), low-density lipoprotein cholesterol (LDL-c) and high-density lipoprotein cholesterol (HDL-c) in liver tissue (*n* = 8). ^a,b^ Different letters represent significant differences at *p* < 0.05.

There were no significant changes following BA treatment regarding TG, TC, LDL-c, and HDL-c in serum (*p* > 0.05, Figure 4). In the liver, Oil Red O staining indicated that low levels of BA inclusion reduced lipid deposition in hepatic tissues compared to the Ctrl and High-BA groups (*p* > 0.05, Figure 5A,B). Furthermore, the TG level in the High-BA group was significantly decreased compared to the Ctrl group (*p* < 0.05, Figure 5C). However, in the Low-BA group, the TG values were decreased (*p* = 0.055, Figure 5C). There were no significant changes in the TC content and lipoprotein in the liver with increasing BA dosage (*p* > 0.05, Figure 5D,E).

### 3.6. Genes Expressions Response to Dietary BAs in the Liver and Ileum

**Figure 6:**
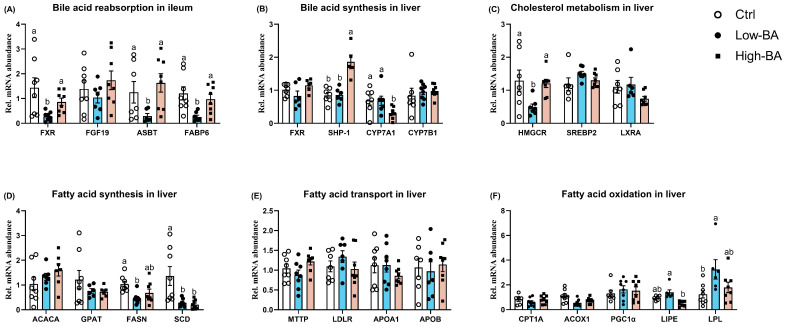
Effects of dietary bile acids (BAs) on the relative mRNA expression of genes related to BA and lipid metabolism in late-phase laying hens. (**A**) The mRNA levels of genes related to bile acid reabsorption in ileum, including farnesoid X receptor (*FXR*), fibroblast growth factor 19 (*FGF19*), apical sodium-dependent bile acid transporter (*ASBT*), and ileum bile acid-binding protein (*IBABP*). (**B**) The genes related to BA synthesis in liver such as *FXR*, small heterodimer partner 1 (*SHP-1*), cholesterol 7α-hydroxylase 1 (*CYP7A1*), and oxysterol 7α-hydroxylase (*CYP7B1*). (**C**) The mRNA abundances of genes related to cholesterol metabolism in liver, including 3-hydroxy-3-methylglutaryl-CoA reductase (*HMGCR*), sterol regulatory element-binding transcription factor 2 (*SREBP2*), and liver X receptors A (*LXRA*). (**D**–**F**) The transcription of genes responsible for fatty acid synthesis, transport, and oxidation in liver. ^a,b^ Different letters represent significant differences at *p* < 0.05 (*n* = 6). *ACACA*, acetyl-CoA carboxylase alpha; *FASN*, fatty acid synthase; *GPAT*, 3-phosphoglycerol acyltransferase; *SCD*, stearoyl-CoA desaturase; *MTTP*, microsomal triglyceride transfer protein; *LDLR*, low-density lipoprotein receptor; *APOA1*, apolipoprotein A1; *APOB*, apolipoprotein B; *PGC1α*, peroxisome proliferator-activated receptor γ Co activator 1-alpha; *CPT1A*, carnitine palmitoyl transferase 1A; *ACOX1*, acyl-CoA oxidase 1; *LIPE*, lipase E, hormone-sensitive type; *LPL*, lipoprotein lipase.

Regarding the BA reabsorption in the ileum, the low-dose BA treatment notably downregulated (*p* < 0.05) the expressions of *FXR*, apical sodium-dependent BA transporter (*ASBT*), and ileum bile acid-binding protein (*IBABP*) when compared to the Ctrl diet, which was similar to the High-BA groups (Figure 6A). Meanwhile, the diet containing high levels of BAs depressed the hepatic BA synthesis, evidenced by decreased mRNA abundances of *CYP7A1* and upregulated the expression of small heterodimer partner 1 (*SHP-1*) in the liver (Figure 6B).

Detection of the gene-related cholesterol metabolism revealed that the supplementation of low BAs reduced (*p* < 0.05) the expression of 3-hydroxy-3-methyl glutaryl coenzyme A reductase (*HMGCR*) relative to the Ctrl groups (Figure 6C). The BA manipulation failed to change the transcription of sterol regulatory element-binding transcription factor (*SREBP*)-2 and nuclear receptor subfamily 1 group H member 3 (*LXRA*) in the liver (Figure 6C). According to the findings in Figure 6D, the supplementation of BAs decreased the mRNA levels of fatty acid synthase (*FASN*) and stearoyl-CoA desaturase (*SCD*) when compared to the Ctrl group. The mRNA abundances of lipid transport genes, including microsomal triglyceride transfer protein (*MTTP*), low-density lipoprotein receptor (*LDLR*), apolipoprotein A1 (*APOA1*), and apolipoprotein B (*APOB)*, were not significantly affected by bile acid treatment (*p* > 0.05, Figure 6E). Furthermore, the Low-BA administration significantly upregulated the expression of lipase E, hormone-sensitive type (*LIPE*) relative to the Ctrl group (*p* < 0.05), and lipoprotein lipase (*LPL*) slightly increased (Figure 6F). There were no significant differences between the Ctrl and High-BA groups in terms of carnitine palmitoyl transferase 1A (*CPT1A*), acyl-CoA oxidase 1 (*ACOX1*), and peroxisome proliferator-activated receptor γ Co activator 1-alpha (*PGC1α*) according to Figure 6F.

## 4. Discussion

With the extension of the egg production period, some adverse impacts have been found in laying hens, including a compromised laying rate, decreased egg quality, excessive fat deposition, and the occurrence of FLHS [1], which are closely linked to animal welfare and human food safety. The supplementation of BAs in HFD was found to improve laying performance and egg quality, and remit liver disease of hens [20], suggesting that the dietary BA supplementation might improve the production performance and lipid metabolism of late-phase laying hens. The outcomes of the present study showed that the dietary BA treatment has no apparent effects on laying performance, whereas it increased the follicle frequency, eggshell weight, and yolk color. Notably, a diet containing 95.01 mg/kg BAs reduced TG deposition in the liver by inhibiting fatty acid synthesis and promoting fatty acid oxidation, which may have a beneficial effect on the liver in late-phase layers.

In practice, commercial laying hens have an extended laying cycle, lasting from 72 weeks up to 80 weeks [21], which is accompanied by a downward trend in the egg production performance. Studies have shown that supplementation of 60 mg/kg porcine BAs increased the laying performance of late-phase hens [18]. However, in the current study, the BA treatment did not seem to change the laying performance, including egg production, egg mass, feed consumption, and feed conversion ratio. Sun et al.’s research results similarly showed that a diet with 100 mg/kg and 200 mg/kg BAs had no significant effect on the production performance [19], suggesting that the effects of BAs on production performance might depend on the dosage, age of laying hens, and duration of feeding. Of note, the production of eggs is closely associated with the development and ovulation of the ovaries, and promoting follicle development can improve the egg production performance of laying hens [23]. It was reported that an increase in the number of small yellow follicles can help to improve the average egg production and egg weight of laying hens [24]. Research has shown that adding BAs to the diet can increase the number of follicles in laying hens, thereby promoting production performance [25]. Our experimental results are consistent with these findings, as the total number of follicles was increased due to the supplementation with BAs. This might increase the possibility of an extended laying cycle in the production of laying hens.

Egg quality, an important parameter for global egg production, depends on many factors, including the diet and age of hens [26,27]. Research indicates that older hens produce eggs with a lower eggshell proportion [28]. It was reported that doses of BAs ranging from 0 to 600 mg/kg had no adverse effects on egg quality [29]. In this study, egg yolk color was increased at 75 weeks with BA supplementation. It is well known that carotenoids play a key role in the deposition of pigmentation in egg yolk, and feeding a diet lacking in carotenoids can cause the yolk or skin of hens to turn white [30]. The addition of porcine BAs increased egg yolk color in this current study might be due to the fact that exogenous BAs may facilitate the absorption of carotenoids, which is consistent with the findings of Sun et al. (2023) [19]. Moreover, the addition of BA supplements contributed to an increase in eggshell proportion at 75 weeks. This is probably the result of promoting calcium absorption, according to a previous study [31]. Taken together, dietary supplementation with BAs enhances yolk color and the eggshell ratio, with no observed adverse effects over the long term.

Serving as a critical regulator, BAs could decrease the content of TC, TG, and LDL-c in serum, leading to reduced abdominal adipose tissue in laying hens [18]. Although no significant effect on the serum lipid metabolism of laying hens, the diet with 100 mg/kg and 200 mg/kg BAs was found to reduce liver lipid deposition and mortality [19]. Similarly, our results show that the dietary BA treatment had no significant effect on serum lipid profile, whereas it decreased the levels of TG in the liver, which was further supported by the quantification results of Oil Red O. This would lead to the vacuolization in liver tissue, i.e., the presence of lipid droplets in the cytoplasm pushes the nucleus of hepatocyte against the cell membrane, indicating fatty degeneration of the organ [5]. In this study, H&E slice staining results indicated that BAs did not reduce the particle size and area of lipid droplet vacuoles. In addition, the serum ALT content was compared and found to be increased in the Low-BA and High-BA groups, suggesting that a low dose of BA supplementation did not impair hepatic health in late-phase laying hens. To explore how the BAs affect the lipid metabolism in the liver, genes related to fat metabolism were detected and showed that the low level of BA treatment reduced the expression of *FASN* and *SCD*, and increased the expression of *LPL*. *FASN* takes charge of the de novo synthesis of fatty acids using acetyl-CoA and malonyl-CoA [32]. *SCD* is the rate-limiting enzyme in the biosynthesis of monounsaturated fatty acids [33,34], which is reported to play an important role in energy metabolism [35]. *SCD1* is a major substrate for TG synthesis, and studies have also shown that *SCD1* knockout mice exhibit reduced hepatic TG accumulation [36]. Moreover, *LPL* mediates the hydrolysis of TG packed in lipoproteins such as chylomicrons and *VLDL*. The inhibition of *LPL* caused a reduction in the lipid droplets and the mRNA expression of lipid metabolism-related genes such as *FASN* [37]. The increased expression of *LPL* and the downregulated abundance of *FASN* and *SCD* in the low-BA group imply that the diet with low doses of BAs reduced lipogenesis and promoted lipolysis, resulting in low fat deposition in the liver. However, the high level of BAs in the diet reduced the TG content in the liver mainly by depressing the synthesis in the liver, evidenced by the decreased mRNA levels of *SCD* in this study. Studies have also shown that the addition of bile acids to broiler diets promotes the expression of genes related to fat metabolism, promotes lipolysis metabolism, and inhibits fat synthesis [38]. Xu et al. (2022) showed that the addition of 900 mg/kg BAs could increase the expression of lipolytic enzymes (*L.PS*, *CPT1*, *ATGL*) and reduce the expression of adipose synthase (*ACC*, *FAS*), thereby improving liver lipid metabolism in fish [39].

Of note, the intestinal reclamation of BAs is crucial for the maintenance of their enterohepatic circulation [40]. In the intestine, *FXR* controls the absorption of BAs through the regulation of expression of four important transporters, including *ASBT*, *FABP6*, and organic solute transporters α (*OSTα*) and β (*OSTβ*). *ASBT*, the major BAs transport system in ileal enterocytes, transports BAs into the ileal enterocyte brush border (apical) membrane, while *FABP6* is expressed in the ileum and shuttles BAs from the apical to the basolateral membrane in the enterocyte [41]. In the present study, the supplementation of low BA but not high BA reduced the expression of *FXR*, *ASBT*, and *FABP6*, indicating that the low dose of BA feeding inhibited the resorption of BAs in the ileum. Previous studies have indicated that an increase in the content of conjugated bile acids (TCDCA, THDCA, THCA, TαMCA) and a decrease in the content of unconjugated BAs (LCA, HCA, HDCA) in the ileum can inhibit the expression of genes related to BAs reabsorption, thereby regulating lipid metabolism in the liver of laying hens under the influence of intestinal microbiota [42]. In addition, *FXR* can be upregulated by BAs in the terminal ileum and further induces the expression of *FGF15*/19, which goes into the liver through the portal vein and inhibits *CYP7A1* expression and hepatic BA synthesis from cholesterol. The downregulated levels of *HMGCR*, a protein-coding gene that catalyzes the rate-limiting step in cholesterol biosynthesis, and comparable *CYP7A1* expression suggested that a low dose of BA supplementation depressed cholesterol biosynthesis, whereas it did not change BA synthesis via the *CYP7A1* pathway. There was another pathway to regulate BA synthesis, where *FGF15*/*19* binds to the heterodimer receptor *FGFR4*/*β-Klotho* in liver cells and regulates BA synthesis [43]. In the liver, the high level of BA treatment activates the *FXR*/*CYP7A1* pathway by upregulating *SHP-1* to suppress BA synthesis in this study. In addition, differences in BA synthesis pathways at high and low doses were also observed in the study by Sun et al. [19]. We cannot explain the difference between low and high BA doses in terms of BA metabolism, and further exploration is required in future studies.

## 5. Conclusions

In summary, dietary supplementation with BAs has no adverse effects on laying performance and hepatic health. The diet containing 95.01 mg/kg of BAs depressed BA resorption in the ileum and hepatic fatty deposition via reducing lipogenesis and promoting lipolysis, which accompanies an increased follicle frequency, yolk color, and eggshell ratio in late-phase laying hens. High levels of BAs (189.99 mg/kg) treatment inhibited hepatic BA synthesis and reduced hepatic TG content, which mainly involves the depression of fatty acid synthesis in the liver. However, further research is needed to understand the mechanisms by which BAs regulate hepatic lipid metabolism in late-phase laying hens.

## Figures and Tables

**Table 1:** Ingredients and nutrient level of the basal diet (as-fed).

Ingredients	Proportion (%)	Nutrient Levels	Proportion (%)
Corn	65.80	ME/(MJ/kg)	2650
Soybean meal	22.00	CP	14.8
Liquid methionine	0.16	Calcium	3.85
Stone powder	9.60	Total phosphorus	0.44
Dicalcium phosphate	0.80	Lysine	0.77
Choline	0.14	Methionine	0.36
Premix ^1^	1.50	Threonine	0.57
Total	100		

^1^ Provided per kilogram of diet: vitamin A 4500 IU; vitamin D 5500 IU; vitamin E, 16 IU; vitamin K 0.5 mg; thiamine, 2.0 mg; riboflavin 5.0 mg; vitamin B6 4.5 mg; vitamin B12 24 mg; Cu (CuSO_4_·5H_2_O) 14 mg; Fe (FeSO_4_·7H_2_O) 85 mg; Zn (ZnSO_4_·7H_2_O) 75 mg; Mn (MnSO_4_·H_2_O) 78 mg; Se (NaSeO_3_) 0.7 mg; I (KI) 0.7 mg; calcium pantothenate 15.0 mg; folate 2.5 mg; biotin 0.15 mg; nicotinic acid 42 mg. ME, apparent metabolism energy; CP, crude protein.

**Table 2:** Primers for quantitative real-time PCR.

Gene ^#^	Gene ID	Primer Sequences (5′-3′)	Product Size (bp)
*ACACA*	NM_205505.2	F: TTGTGGCACAGAAGAGGGAAT	143
		R: AGTGAGGTCAAAGTTCCGCA	
*FASN*	NM_205155.4	F: GCTAAGATGGCATTGCACGG	135
		R: TCCATTCAGTTCCAGACGGC	
*GPAT*	NM_001004401.2	F: TGGACGTGCCCCATGTGAT	101
		R: TGCCTCGGATGACTCTCCAT	
*MTTP*	NM_001109784.3	F: GCAGATGGACAGAGTTGGCT	93
		R: TTCCCTCTCCTCGCAGTGTA	
*LDLR*	NM_204452.1	F: TTCGAGGACTCCGTGTTCTG	100
		R: GCAGAGATTCGGCCACGAC	
*APOA1*	NM_205525.5	F: TGAGGACATGGCTCCCTACTA	141
		R: CACTTGGCAGAGAACTGGTCC	
*APOB1*	NM_001044633.2	F: GCAGCTTTGCTCATCGTGAC	119
		R: AACGTCAGCAAATGTTGGGC	
*LXRA*	XM_040700552.2	F: GTATATGCGCCGCAAGTGTC	76
		R: CAGAACATACTGCTCCCGCA	
*SREBP2*	XM_040660556.2	F: CTCGTGAATGGTGTGATCGTCCTC	112
		R: GCTTGCGGTGCCTCCAGAAC	
*HMGCR*	NM_204485.3	F: AGGACCTGTTGTAAGGCTGC	124
		R: TAGGCGGGCAAACCTACTTG	
*CYP7A1*	NM_001001753.2	F: GCTCCGCATGTTCCTGAATG	99
		R: ATGGTGTTAGCTTGCGAGGC	
*CYP7B1*	XM_025147742.3	F: TGCGTGACGAGATTGACCAT	121
		R: TCGTTTAAGGCGCTCTCCAG	
*FXR*	NM_001396910.1	F: CAACCTGGGCTTCTACCCTC	145
		R: GTGGCCCAGTCTAGGCTTTT	
*PGC1α*	NM_001006457.2	F: GAGTGACATCGAGTGTGCTG	143
		R: ACTGGTCGCTGTACCACTTG	
*CPT1A*	XM_046918285.1	F: GCTCACTACCGAGACATGGG	92
		R: GACCGGACGGTTTCAGTTCT	
*ACOX1*	NM_001006205.2	F: AGGAGATCGAGGCCTTAGTGA	89
		R: GGCTTGTTCATAGCGTTGGC	
*LIPE*	XM_040657096.1	F: CCTTCTTCCTCACCACGGAC	107
		R: TTGGAGGTGTCTCAAAGGGC	
*SCD*	NM_204890.2	F: TTAGGGCTCAATGCCACCTG	90
		R: GTTCTCCCGTGGGTTGATGT	
*FGF19*	NM_204674.3	F: CCGCTGTCTCACTTCTTACCC	120
		R: CGTTTCGAGAGGCGATGAGTA	
*ASBT*	NM_001319027.2	F: GCTGTGGTTGGGGGAATACT	137
		R: CTGCTCCAAGACAGACCAGC	
*FABP6*	NM_001277700.2	F: GGACGCACCACGACTAATTC	100
		R: TCCCACCTTCCATTTTGACTGT	
*SHP-1*	NM_001030893.3	F: ACGCACTGAGCTACAGACAC	105
		R: AGGGAGCTTTCCAGACATGC	
*LPL*	NM_205282.2	F: TCGCAGCATTGGGATTCAGA	109
		R: TTCAGCAATCAGGCGGAGAG	
*β-actin*	NM_205518.1	F: GTCCACCGCAAATGCTTCTAA	78
		R: TGCGCATTTATGGGTTTTGTT	

^#^ *ACACA*, acetyl-CoA carboxylase alpha; *FASN*, fatty acid synthase; *GPAT*, 3-phosphoglycerol acyltransferase; *MTTP*, microsomal triglyceride transfer protein; *LDLR*, low-density lipoprotein receptor; *APOA1*, apolipoprotein A1; *APOB*, apolipoprotein B; *LXRA*, liver X receptor A; *HMGCR*, 3-hydroxy-3-methylglutaryl-CoA reductase; *CYP7A1*, cholesterol 7α-hydroxylase; *CYP7B1*, oxysterol 7α-hydroxylase; *FXR*, farnesoid X receptor; *PGC1α*, peroxisome proliferator-activated receptor γ Co activator 1-alpha; *CPT1A*, carnitine palmitoyl transferase 1A; *ACOX1*, acyl-CoA oxidase 1; *LIPE*, lipase E, hormone-sensitive type; *SCD*, stearoyl-CoA desaturase; *FGF19*, fibroblast growth factor 19; *ASBT*, apical sodium-dependent bile acid transporter; *FABP6* (*IBABP*), ileum bile acid-binding protein; *SHP-1*, small heterodimer partner 1; *LPL*, lipoprotein lipase; *SREBP2*, sterol regulatory element-binding transcription factor 2; *β-actin*, reference genes.

**Table 3:** Effects of dietary treatments on egg quality in late-phase laying hens.

Item	Ctrl	Low-BA	High-BA	*p*-Value
Egg-shaped index	1.31 ± 0.01	1.31 ± 0.01	1.31 ± 0.01	0.950
Eggshell properties				
Relative weight, %	12.85 ± 0.22 ^b^	13.46 ± 0.14 ^a^	13.46 ± 0.21 ^a^	0.030
Thickness, mm	0.32 ± 0.003	0.33 ± 0.002	0.32 ± 0.003	0.137
Strength, N	37.03 ± 1.06	39.51 ± 1.00	38.13 ± 1.20	0.284
Yolk properties				
Relative weight, %	26.72 ± 0.34	27.07 ± 0.27	26.38 ± 0.31	0.260
Yolk index	42.14 ± 0.45	41.49 ± 0.52	42.81 ± 0.54	0.189
Yolk color	11.89 ± 0.11 ^b^	12.17 ± 0.07 ^a^	12.13 ± 0.09 ^ab^	0.045
Protein properties				
Protein height, mm	5.77 ± 0.25	5.28 ± 0.14	5.37 ± 0.18	0.142
Haugh unit	73.36 ± 2.10	71.97 ± 1.25	70.96 ± 1.44	0.241

Values mean ± standard error (*n* = 8). ^a,b^ Different letters in the same column represent the significant difference at *p* < 0.05.

**Table 4:** Effects of dietary treatments on organ index in late-phase laying hens.

Item	Ctrl	Low-BA	High-BA	*p*-Value
Body weight, kg	2.10 ± 0.07	2.13 ± 0.06	1.96 ± 0.05	0.165
Absolute weight, g				
Heart	7.06 ± 0.32	8.07 ± 0.72	7.23 ± 0.31	0.464
Liver	33.51 ± 2.20	34.20 ± 1.99	32.29 ± 1.33	0.744
Spleen	2.17 ± 0.21	2.10 ± 0.12	2.05 ± 0.09	0.984
Pancreas	3.76 ± 0.29	3.69 ± 0.21	3.16 ± 0.13	0.127
Ovarian	41.43 ± 3.63	37.45 ± 1.62	43.43 ± 2.76	0.292
Relative weight, % body weight				
Heart	0.34 ± 0.01	0.37 ± 0.03	0.36 ± 0.01	0.354
Liver	1.58 ± 0.06	1.58 ± 0.06	1.52 ± 0.06	0.645
Spleen	0.10 ± 0.007	0.10 ± 0.007	0.10 ± 0.004	0.886
Pancreas	0.16 ± 0.01	0.17 ± 0.01	0.16 ± 0.01	0.681
Ovarian	2.19 ± 0.22	1.78 ± 0.13	2.25 ± 0.13	0.102
Absolute intestinal length, cm				
Duodenum	22.55 ± 0.90	24.56 ± 0.60	22.50 ± 0.85	0.150
Jejunum	44.90 ± 2.93	46.10 ± 2.12	50.60 ± 2.38	0.253
Ileum	42.66 ± 1.41	37.78 ± 1.58	41.90 ± 2.12	0.132
Relative intestinal length, cm/kg body weight				
Duodenum	10.19 ± 0.38	11.08 ± 0.50	10.84 ± 0.47	0.358
Jejunum	20.26 ± 1.20	21.23 ± 1.12	23.96 ± 0.92	0.070
Ileum	19.31 ± 0.64 ^ab^	17.26 ± 0.77 ^b^	21.05 ± 1.22 ^a^	0.028

Values mean ± standard error (*n* = 8). ^a,b^ Different letters in the same column represent the significant difference at *p* < 0.05.

## Data Availability

The original contributions presented in the study are included in the article; further inquiries can be directed to the corresponding author.
